# Equine syndromic surveillance in Colorado using veterinary laboratory testing order data

**DOI:** 10.1371/journal.pone.0211335

**Published:** 2019-03-01

**Authors:** Howard Burkom, Leah Estberg, Judy Akkina, Yevgeniy Elbert, Cynthia Zepeda, Tracy Baszler

**Affiliations:** 1 The Johns Hopkins University Applied Physics Laboratory, Laurel, Maryland, United States of America; 2 Center for Epidemiology and Animal Health, US Department of Agriculture, Ft. Collins, Colorado, United States of America; 3 Veterinary Diagnostic Laboratory, College of Veterinary Medicine and Biomedical Sciences, Colorado State University, Fort Collins, Colorado, United States of America; New York City Department of Health and Mental Hygiene, UNITED STATES

## Abstract

**Introduction:**

The Risk Identification Unit (RIU) of the US Dept. of Agriculture’s Center for Epidemiology and Animal Health (CEAH) conducts weekly surveillance of national livestock health data and routine coordination with agricultural stakeholders. As part of an initiative to increase the number of species, health issues, and data sources monitored, CEAH epidemiologists are building a surveillance system based on weekly syndromic counts of laboratory test orders in consultation with Colorado State University laboratorians and statistical analysts from the Johns Hopkins University Applied Physics Laboratory. Initial efforts focused on 12 years of equine test records from three state labs. Trial syndrome groups were formed based on RIU experience and published literature. Exploratory analysis, stakeholder input, and laboratory workflow details were needed to modify these groups and filter the corresponding data to eliminate alerting bias. Customized statistical detection methods were sought for effective monitoring based on specialized laboratory information characteristics and on the likely presentation and animal health significance of diseases associated with each syndrome.

**Methods:**

Data transformation and syndrome formation focused on test battery type, test name, submitter source organization, and specimen type. We analyzed time series of weekly counts of tests included in candidate syndrome groups and conducted an iterative process of data analysis and veterinary consultation for syndrome refinement and record filters. This process produced a rule set in which records were directly classified into syndromes using only test name when possible, and otherwise, the specimen type or related body system was used with test name to determine the syndrome. Test orders associated with government regulatory programs, veterinary teaching hospital testing protocols, or research projects, rather than clinical concerns, were excluded. We constructed a testbed for sets of 1000 statistical trials and applied a stochastic injection process assuming lognormally distributed incubation periods to choose an alerting algorithm with the syndrome-required sensitivity and an alert rate within the specified acceptable range for each resulting syndrome. Alerting performance of the EARS C3 algorithm traditionally used by CEAH was compared to modified C2, CuSUM, and EWMA methods, with and without outlier removal and adjustments for the total weekly number of non-mandatory tests.

**Results:**

The equine syndrome groups adopted for monitoring were abortion/reproductive, diarrhea/GI, necropsy, neurological, respiratory, systemic fungal, and tickborne. Data scales, seasonality, and variance differed widely among the weekly time series. Removal of mandatory and regulatory tests reduced weekly observed counts significantly—by >80% for diarrhea/GI syndrome. The RIU group studied outcomes associated with each syndrome and called for detection of single-week signals for most syndromes with expected false-alert intervals >8 and <52 weeks, 8-week signals for neurological and tickborne monitoring (requiring enhanced sensitivity), 6-week signals for respiratory, and 4-week signals for systemic fungal. From the test-bed trials, recommended methods, settings and thresholds were derived.

**Conclusions:**

Understanding of laboratory submission sources, laboratory workflow, and of syndrome-related outcomes are crucial to form syndrome groups for routine monitoring without artifactual alerting. Choices of methods, parameters, and thresholds varied by syndrome and depended strongly on veterinary epidemiologist-specified performance requirements.

## Introduction

The global animal health stakeholder community has increasingly embraced syndromic surveillance for routine situational awareness of animal population health. These efforts are attracting attention and funding beyond the agricultural community because of the One Health initiative and because of multiple programs aimed at integrated infrastructure surveillance [1–6]. These programs enhanced interest in livestock monitoring combined with advances in data management technologies and veterinary informatics is producing novel data streams of varying quality and utility. The current effort was the first step in a project aimed at identifying best practices for routine syndromic surveillance using veterinary diagnostic laboratory testing orders.

### Ongoing animal health monitoring activities

This paper documents the expansion of animal health surveillance activities at the United States Department of Agriculture (USDA), Animal and Plant Health Inspection Service (APHIS), Veterinary Services (VS), Center for Epidemiology and Animal Health (CEAH). Epidemiologists in the Risk Identification (RI) group of CEAH conduct regular monitoring of animal health data with the objective to build and maintain a syndromic surveillance system that utilizes non-traditional data (typically collected for other purposes) to augment emerging disease surveillance and inform stakeholders about comprehensive animal health beyond presence or absence of specific diseases. Stakeholders include animal commodity organizations, veterinarians, and State and Federal government animal health agencies. The main purpose of the monitoring is to identify abrupt increases in selected data indicators that may signal emerging disease outbreaks or other health concerns. Data analyses may also identify health trends, provide supporting evidence for freedom from disease, and support risk factor analysis [1].

### Requirements for meeting current and future monitoring needs

Strategic plans to increase the number of species, health issues, and data sources monitored require both broadening and deepening of current capabilities. The broadening entails a) expanded collaboration with government and private agencies with related interests, b) routine access to more extensive data sources of varying clinical specificity, and c) informatics systems to automate routine monitoring tasks and to present results promptly and clearly. The deepening requires understanding of novel data sources and the utility of their elements for various animal health monitoring purposes, and also effective and practical analytical tools to extract and concisely summarize key information in these data.

The RI group currently analyzes data streams to monitor animal populations for indirect evidence of known and unknown diseases with limited signal investigation resources and no gold standards for signals of interest. Future analytic needs depend on the richness of available data and prioritized monitoring goals; current and projected needs include syndromic categorization of data to detect both known and unknown health threats, best-practice monitoring of syndromic streams, space-time anomaly detection, and fusion of evidence from multiple sources.

#### Near-term approach

Progress toward capability expansion must be planned within human, informatics, and technological resource constraints. The current project explored the feasibility and value of monitoring testing order data from the Colorado State University (CSU) Veterinary Diagnostic Laboratories (VDL). Collaboration in this project involved veterinary epidemiologists and informaticists, laboratorians, horse health experts in VS and at CSU, and statistical analysts and system developers from the Johns Hopkins University Applied Physics Laboratory (JHU/APL).

The approach was to monitor time series of weekly counts of groups of tests corresponding to syndromes, similar to the syndromic approach applied by the RI group to other national and regional data sources. The first development tasks were to create syndrome groups for the VDL lab data and to customize algorithms for those syndromes within the constraints of weekly monitoring and system limitations of the analysis tools already in use. Objectives herein are to describe how these tasks were addressed, some of the issues faced, and how collaborators managed them, for the benefit of other groups that receive complex surveillance-related data with similar tasking. Discussion and results are limited to the first expansion project, restricted to surveillance of equine diseases in Colorado. We focused on equine diseases to complement a concurrent pilot project that collected and monitored syndromic data from equine veterinary practitioners in Colorado. In addition to description of the syndrome development process, the syndrome classification rules and the algorithms chosen may be useful to others, depending on their own data environment and constraints.

## Materials and methods

### Source dataset

The study dataset was a collection of 12 years of equine laboratory test records from the Veterinary Diagnostic Laboratories (VDL) of Colorado State University [7]. The dataset was extracted from the CSU VDL Laboratory Information Management System (LIMS). The data fields requested were selected based on their ability to a) describe a specific animal health event that resulted in a request for diagnostic testing, b) provide spatio-temporal information related to the animal health event, and c) characterize the observed disease syndrome associated with the animal health event. While geographic fields such as the zip code and city of the submitter and zip code of the owner were included, the data provided had no personally identifiable information (PII) on the testing order submitters or owners of the animals to be tested. Each dataset row could represent one of multiple primary events including ‘lab submission received’, ‘test order logged’, and ‘test result obtained’. The initial objective was to restructure each dataset row into information that could be useful for identifying syndrome-specific testing ordered for individual horses (cases) as early as possible.

### Case definition and syndrome classification approach

Cases within each syndrome category were identified as individual horses (defined by a unique animal ID) included in a laboratory submission (defined by a unique accession ID) comprising of one or more test orders satisfying case definition rules. The syndrome classification procedure (including application of the case definition rules) and findings are provided in the Methods and Results sections. During case identification, a laboratory submission for a single animal could be assigned to more than one syndrome, resulting in a single animal being represented as multiple cases in different syndromic categories. For example, “Aspergillis AGID” testing for a horse may be classified as a case in both “Respiratory” and “Systemic Fungal” syndromes. Following case identification, weekly case counts were summed over a 7-day (Sunday to Saturday) time period based on the laboratory submission date. Case definition rules were developed to prevent counting a case more than once within a syndrome and to avoid counting test orders unrelated to sick animals.

### Data content analysis and key information fields

**Fig 1:**
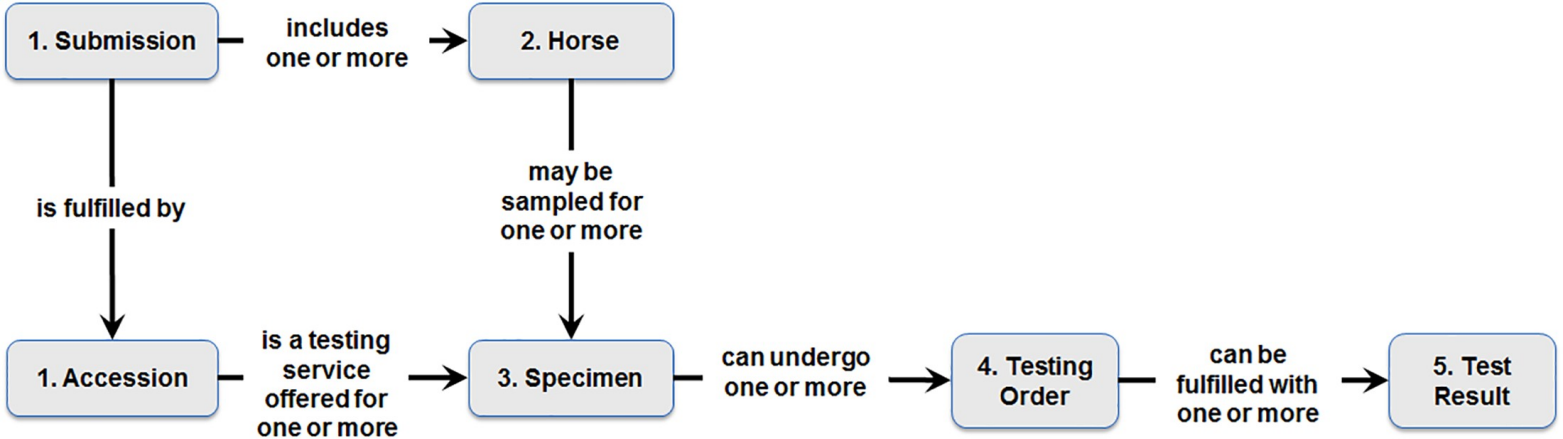
Formation of veterinary laboratory information.

The processes and data fields used to convert the test record data into syndrome case counts for routine monitoring are performed using Microsoft ACCESS. Fig 1 depicts the steps in the data formation process.

Steps in the data formation process were:

1. Accession/submission. A lab submission is most commonly generated from a veterinarian (test order submitter) visiting a site where horses live. Submissions may also originate when horses are transported to a private practice, a tertiary hospital, or an event such as a race or competition. The date the submission was received by the lab is used as the date each case was detected. The testing battery ordered is a data field sometimes provided at the accession level and is used to identify and classify syndrome cases. Though the RI epidemiologists consider the test battery as one of the clearest indications of the submitting veterinarian’s concerns, the data field for test battery was completed in less than 4% of submissions for the data available.
2. Horse: Each horse has a unique identifier (within the context of the accession ID). Multiple horses may be included in the testing submission. The details about the horse tested are provided including breed, sex, age, indicators for identifying horse subjects for research or teaching hospital patients, and clinical history (if available), All animals specified as ‘Equine’, and not as research subjects, are identified as potential cases. If provided, the animal’s owner location information (i.e. zip code) is used as the primary indicator of the case location. No additional information is provided about the animal’s owner. The submitter location information is provided if available (i.e. zip code, city, state), and used as an indirect indicator of the horse location if the animal owner location is not provided.
3. Specimen: All specimens included in each accession are uniquely identified with the lab assigned specimen ID. In the case definition rules described below, the type of specimen (e.g. serum) is used to assist classifying cases if the test battery and test name are not considered sufficient.
4. Testing Order: All testing orders placed for each specimen are uniquely identified with a standardized test name assigned by the lab receiving staff. New and updated testing orders arriving each week for inclusion in the database are used to update the testing information associated with each specimen.
5. Test Result: All results associated with each testing order are uniquely identified with the ‘analyte measured’ and the result value. New and updated test results arriving each week are used to update the result information associated with each testing order.

### Formation and refinement of case definition rules

**Fig 2:**
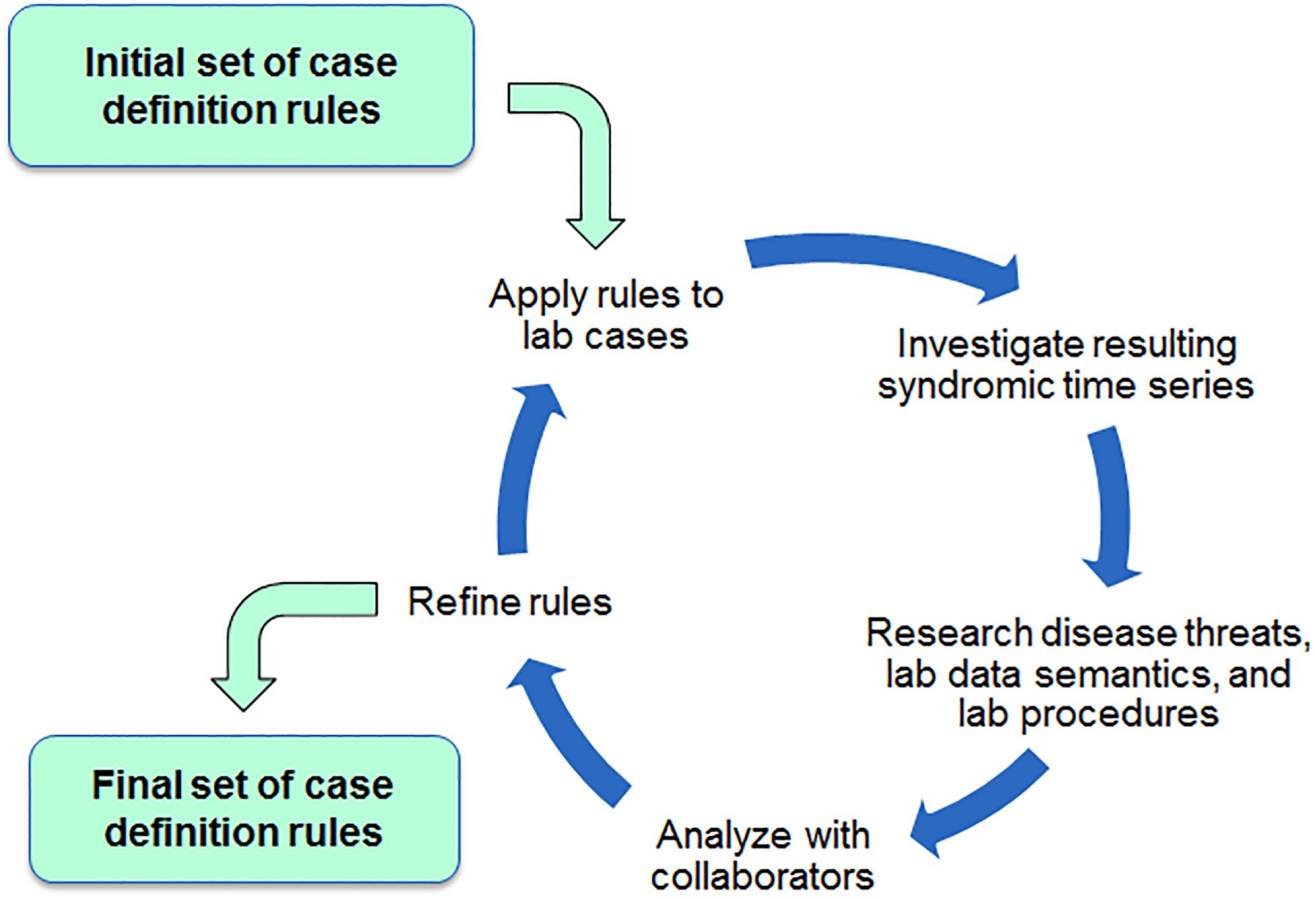
Schematic depicting the case definition refinement process.

The syndrome categories were intended to classify horses displaying clinical signs associated with a body system, with a focus on infectious diseases. Because the submitting veterinarian’s original intent for each lab testing order was rarely submitted for inclusion in the LIMS, the case definition rules were based on likely clinical signs suggested by the testing ordered. The approach conducted for developing the case definition rules is shown in Fig 2, illustrating the iterative collaboration among APHIS epidemiologists and veterinarians, CSU VDL laboratorians, and JHU/APL statistician analysts.

Trial case definitions were formed based on consultation with VS horse health experts, investigation of test use, and published literature. We formed time series using weekly case counts assigned to these syndromes and analysed them considering mean and variance of syndrome count levels, year-to-year trend consistency, frequency of data spikes, and outliers related to certain test/specimen types and a few health events known from RI group experience or published literature. The analysis led to further consultations with CSU laboratorians regarding the data field usage, lab work flow, and CSU veterinary hospital protocol. Based on these consultations, we revised the case definition rules, formed new time series, and repeated the analysis. Ultimately, we converged on the case definition rules explained in the Results section and listed in S1 and S2 Tables.

### Algorithm test and evaluation

#### Algorithms selected

We originally intended to perform spatial cluster detection, but the quality of the spatial data precluded this effort. The only location fields available in the test order records were submitter and owner zip codes. Owner zip codes were present in only a minority of the test orders, and when provided, many submitter zip codes were located far from these owners (the owner’s zip codes are assumed to match the majority of residence zip codes of the horses). Anomalous clusters would have been biased and unrepresentative of spatial groups of sick animals, so we restricted candidate alerting algorithms to temporal methods.

Using the syndromic time series from the historical dataset, a set of temporal algorithms were compared to the method adopted by the RI group in 2010 for slaughter condemnation syndrome monitoring. This traditional method was the CDC EARS C3 algorithm with parameters and thresholds chosen to fit syndromes for weekly monitoring [7]. The additional methods were introduced to seek customized alerting performance relative to the newly developed lab-based syndromes. Candidate methods tested were adaptations of control charts and simple models previously published for prospective health surveillance. These candidates include the modified C2 method [8], adaptive versions of the cumulative summation (CuSUM) and exponentially weighted moving average (EWMA) charts [9], the temporal scan statistic Gscan of Naus and Wallenstein [10], and the CDC Historical Limits method [11]. These methods were tested with and without features for outlier removal and for denominator adjustment to the total weekly number of tests.

#### Algorithm evaluation

Practical performance evaluation of alerting algorithms requires results of the algorithms applied to authentic data labelled with outbreak and non-outbreak intervals. Such data are needed for computation of sensitivity, positive predictive value, timeliness, and other detection metrics. However, literature, institutional, and other searches yielded only a few equine outbreaks of common diseases that were represented in available data, and no outbreaks of rare diseases. Furthermore, APHIS monitors animal populations for known and unknown, seasonal and nonseasonal diseases. Rather than extrapolate from sparse published guidance, we adopted a simulation approach informed by the scientific knowledge and animal health monitoring experience of the RI group epidemiologists that would be using the algorithms each week.

We measured algorithm detection performance with many repeated trials, each adding a plausible set of syndrome counts to the historical data. We then compared how well the candidate algorithms detected the simulated signals. For this purpose, we adopted an outbreak effect simulation scheme used in other analytic development efforts based on randomized but realistic injected target signals [12–14]. We constructed a Matlab testbed for sets of 1000 statistical trials and applied a stochastic injection process assuming lognormally distributed incubation periods to choose an alerting algorithm with desired values of sensitivity and alert rate [12]. For each chosen syndrome, the RI group provided incubation period distributions for simulated target signals based on published information.

For algorithm performance metrics, they also provided maximum background alerting rates and minimum sensitivity values. Needing a quantitative basis to compare how well algorithms detect target signals, we chose to use requirements from the experienced RI group staff that must live with the surveillance system rather than employ estimates based on limited literature or derived by analysts lacking domain knowledge.

The performance requirements derived from operational experience and judgment were: 90% sensitivity for most syndromes, elevated to 95% for neurological and tickborne syndromes, while maintaining alert rates of at least one but at most eight weekly signals per year. Along with the Matlab testbed, we constructed an EXCEL analytic visualization tool for collaborative evaluation, similar to the tool used in the RI group since 2010. In practice, algorithms are implemented in Visual Basic code in the ACCESS database, and Tableau software is used to display algorithm results.

## Results

The overall monitoring goal is to detect periods of abnormally high numbers of cases within each syndrome. The technical approach had three principal components: development of case definitions for syndromes, applying the definitions to identify cases, and derivation of a representative set of algorithms and associated parameters and thresholds for routine statistical alerting practice (i.e. development of monitoring algorithms). Each component required iterative collaboration. Exploratory analysis, stakeholder input, and the discovery of laboratory data management and workflow details were needed to modify the case definitions, excluding the testing for reasons other than current observed illness (e.g. regulatory testing, import/export testing) to avoid irrelevant/excessive alerting and masking of signals of genuine interest. Customized statistical detection methods were sought for effective monitoring based on specialized laboratory information characteristics and on the likely presentation and animal health significance of diseases associated with each syndrome. The collaboration to develop the syndromes and select the algorithms required nine months, with weekly or biweekly calls among the authors to formulate and revise syndrome groups and several meetings of the APHIS RI team with CSU laboratorians.

### Syndrome categories selected for monitoring

Eight syndrome categories were chosen for routine monitoring. The most common type is represented as a syndrome of clinically observable signs, often associated with a body system:

1 Abortion/Reproductive (Abortion/Repro)
2 Diarrhea/Gastrointestinal (Diarrhea/GI)
3 Neurologic
4 Respiratory
5 Sudden Death

Some outcomes of concern were considered better classified by disease type as suggested by the testing ordered:

6 Tickborne (e.g. Lyme disease, anaplasmosis)
7 Systemic Fungal (e.g. aspergillosis, coccidioidomycosis)

Lastly, counts of necropsy procedures, excluding those for fatal racetrack injuries, are monitored to enable detection of rare or novel conditions that may be missed by other surveillance categories:

8 Necropsies

### Case definition rules

Most criteria in the case definitions rules were derived from the test order name and specimen type data fields. We analyzed time series of weekly counts of cases included in candidate syndrome groups and conducted an iterative process of data analysis and veterinary consultation for case definition rule refinement. Throughout the iterative process, we identified test order names that should be routinely excluded so that derived syndromes would include only tests that would be ordered for animals suspected of having infectious diseases. Some tests that are routinely associated with clinical signs unrelated to diseases of concern were similarly excluded. Some examples of these tests include organ function tests, endocrine tests and many types of histopathology. Tests representing national or state surveillance programs (e.g., tests for Equine Infectious Anemia) were not included because they are not usually performed on animals for diagnostic purposes, and tests representing the CSU Veterinary Teaching Hospital policies were excluded, such as mandatory fecal testing of hospitalized animals. Some test names were created by the lab to represent specialized billing, and orders with these test names were also excluded.

This iterative development of case definitions produced a rule set in which lab testing information is classified into syndromes using only test order name when possible; but many situations required further information. For example, a pathogen that may cause several syndromic presentations may be categorized based upon the specimen type or related body system of the specimen tested in addition to the test name. One hundred ninety-four unique test order names were identified for horses in the data set. The rules for syndrome categorization followed five general guidelines described below.

**Testing Batteries**: A submitter may specify a battery of tests when placing a laboratory submission. This battery name is a selection from a fixed list and is stored at the accession level in the CSU LIMS. It is associated with all animals and animal groups included in the submission. Though supplied in only 4% of recent data, this name, when available, is the most direct indication of the submitter’s clinical concerns. In the available data, each non-blank battery name contained either the term “diarrhea” or “abortion”, and the test was assigned accordingly to the Diarrhea/GI or the Abortion/Repro syndrome without consideration of other data fields.

**Test Order Name**: In the absence of a battery name, the test name alone is often sufficient for syndrome assignment. Some test names refer to a specific infectious disease or pathogen, and a further subset of these diseases typically present with clinical signs associated with one syndrome. The Test Name field is a standardized fixed field. The rule in this situation is to assign the syndrome based on S1 and S2 Tables.

**Test Order Name and Specimen**: Some tests are ordered for suspected diseases with multiple syndromic presentations. Other tests represent broad infectious disease categories, such as bacterial culture. To resolve the syndromic classification of test names that cannot be mapped directly, we used the semi-standardized ‘Specimen’ data field. For this field, the data entry staff is instructed to use pre-defined types as much as possible. The pre-defined fields do not all directly correspond to sample types given on the CSU VDL website. If an appropriate specimen type cannot be found in the existing list, the data entry person will type it in (free text). The specimen type entered may be generic (e.g. biopsy, swab) if not provided on the lab submission form. We grouped the specimen types of interest into one or more categories and/or body system groups. The specimen categories included are serum, blood, nasal swab, csf (cerebrospinal fluid), fetal tissue, and fungal skin. Specimen body systems included are Digestive, Nervous, Reproductive, and Respiratory. We considered mapping these specimen groupings directly to syndromes regardless of the test name but rejected such mappings as too coarse because they would ignore details and conventional application of individual tests. Instead, we formed rules based on combinations of test name and either specimen body system (i.e., body system most represented by specimen submitted for testing) or specimen category (i.e., tissue type of specimen submitted for testing), including rules of exclusion for some tests. S2 Table lists these combinations and exclusions and was the prescriptive basis for syndrome assignment when the test battery name or test name alone was inconclusive. The tables are implemented as three rule types:

TestNameandSpecimenBodySystem=>Syndrome

TestNameandSpecimenCategory=>Syndrome

TestNameand(NOT(SpecimenBodySystem(s))and/orNOT(SpecimenCategory(s)))=>Syndrome

During rule formation and refinement, assumptions were made on the use of the different available tests for the same pathogen, samples that would typically be submitted, and typical disease presentation. Some of these assumptions will require further validation through real-time monitoring of the data.

### Summary of case definition rules

The final detailed rule set we adopted for syndrome classification is presented in two supplementary tables. S1 Table contains the general syndrome mapping table with columns for syndrome, test name, specimen body system, and specimen category. For rows where the specimen type or category is not blank, both test name and specimen were used to determine a syndrome. S2 Table is provided for determination of the specimen type or category from the text in the Specimen field. We used the rule set defined by these tables to identify cases that were classified into one or more of the eight selected syndromes.

One third (65/194) of the equine tests in the dataset were categorized into one or more syndromes. Remaining tests were excluded because they did not reflect diseases readily transmitted among animal populations, mainly including cultures without indicative specimen types, blood chemistry and hematology panels, antibiotic sensitivity, cytology, fluid analysis, histopathology, and other general test types. Three-fourths (49) of these 65 tests were assigned to a syndrome based on the test name alone. The remaining 16 tests required more complex rules based on the submitted specimen type. Accuracy of these rules is limited by the information available in the test records. Some cases will be erroneously classified as syndrome cases when tests are requested for reasons other than clinical illness, such as for a research study not indicated by the appropriate data flag, or when a clinician is checking titres prior to vaccination. Other cases may not be correctly assigned to syndromes if the specimen type is not specific enough. The qualification of records for indication of likely clinical illness and the subsequent case definition rules proved essential in obtaining syndrome counts representative of current equine population health. Inclusion of the >80% of tests deemed unrelated to current illness trends would likely bias routine health monitoring. Among 45,568 equine tests in the years 2012–2014, only 7,031 (15.5%) were classified into syndromes, with syndromic totals of 2065 (12.7%) in 2012, 2934 (17.6%) in 2013, and 2032 (16.1%) in 2014. Table 1 below describes the two most common tests contributing to each syndromic classification in the years 2012–2014. From this table, the Abortion/Reproductive and relatively rare Sudden Death, Tickborne, and Systemic Fungal syndromes are dominated by one or two tests with at least 80% representation, while the more common syndromes have more variety among categorized tests. `

**Table 1:** Percentages of each syndrome among syndromic test orders (in parentheses), and percentages of orders of top two tests among all orders mapped to each syndrome.

Syndrome (with % of 23,478 syndromic test orders)	Test	Percentage within syndrome
Abortion/Reproductive (21.6%)	Aerobic Culture (reproductive specimen types)	56%
	Endometrial Biopsy	24%
Diarrhea/Gastrointestinal (28.7%)	Clostridium Fecal Culture	42%
	Rotavirus ELISA	13%
Neurologic (9.1%)	Equine West Nile Virus IgM ELISA	36%
	Rabies fluorescent antibody test	27%
Respiratory (28.4%)	Aerobic Culture (respiratory specimen types)	16%
	Streptococcus equi—PCR	15%
Sudden Death (2.6%)	Selenium (Hydride FAAS)	96%
	Bacillus anthracis (Anthrax) real-time PCR	4%
Tickborne (0.1%)	Ehrlichia spp/Anaplasma/Neorickettsia/Wolbachia—PCR	50%
	Tick Panel-Ehrlichia Anaplasma Lyme ELISA	50%
Systemic Fungal (0.2%)	Grocott’s methenamine silver (GMS) stain	67%
	Fungal Culture (not on skin specimen types)	24%
Necropsies (9.4%)	Necropsy & Histopathology (at the CSU Vet Teaching Hospital)	46%
	Necropsy Gross Examination Only	20%

### Algorithm evaluation results

Table 2 presents recommended alerting algorithms, baseline lengths, and algorithm thresholds for each requested combination of syndromes and signal types. An alert is issued when an algorithm value exceeds the threshold. These results were derived from repeated comparative simulation runs. Algorithms, baselines, and thresholds were jointly varied in these runs, and the tabulated values are combinations that best met the required sensitivity and recurrence criteria under typical syndrome incidence patterns.

**Table 2:** ummary of algorithm recommendations for each syndrome and each signal type of concern.

Data and Weekly Count Percentiles and Standard Deviations					Method Details				Detection Performance		
Syndrome	50%	75%	99%	Std. Dev.	Signal Type	Algorithm	Baseline (weeks)	Threshold	Detectable Total Inject Count	Sensitivity	Recurrence (wks)
Abortion/Repro	7.0	15.0	38.9	9.6	1-week spike	modified C2	8	2.5	30	0.93	17.47
Diarrhea/GI	2.0	3.0	11.0	2.4	1-week spike	modified C2	16	2.0	10	0.93	17.76
Necropsy	4.0	5.0	11.0	2.4	1-week spike	modified C2	16	2.5	10	0.93	53.27
Neurological	1.0	4.0	59.9	8.5	1-week spike	modified C2	8	3.0	20	0.93	15.23
Neurological					90% in 8 wks	CuSUM	8	3.5	30	0.90	8.39
Respiratory	8.0	12.8	79.5	14.6	1-week spike	modified C2	8	2.0	30	0.83	14.49
Respiratory					90% in 6 wks	CuSUM	16	2.5	100	0.90	10.98
Sudden Death	1.0	1.0	6.0	1.4	1-week spike	Gscan	8	3.0	10	1.00	21.54
Systemic Fungal	0.0	0.0	3.0	0.7	90% in 4 wks	Gscan	8	2.5	10	0.93	60.30

The table contains three sections for syndromic time series description, details of preferred alerting methods, and detection performance for each syndrome and each signal type indicated by the epidemiologists. The Detectable Total Inject Count column gives the weakest, i.e. most challenging, injected signal size for which the criteria were satisfied.

The time series description section shows the 50^th^, 75^th^, and 99^th^ percentiles and standard deviations of each series of 610 weekly syndrome counts—nearly 12 years of data. The complete time series are available in S3 Table. Note that data scales range from medians of zero and one count per week for the systemic fungal and sudden death syndromes, to over five per week for the abortion/repro and respiratory syndromes.

Table 2 gives the preferred algorithm for each combination of syndrome and signal type with the number of baseline weeks and the alerting threshold that gave the best detection performance relative to the RI group-provided sensitivity and alert rate requirements. We used the *recurrence interval*, or expected number of weeks between alerts, to express the trade-off between sensitivity and the burden of alert investigation. We avoided explicit specificity claims because specificity calculations require the number of true negatives TN, i.e. the true number of weeks with no outbreak. Published specificity values using authentic background data are exaggeratedly high in the surveillance context because most outbreaks are unreported, so TN is inflated.

In consideration of these results, it is important to keep in mind that for the goal of choosing the relatively best algorithm, we kept increasing the strength of the injected signal until one of the algorithms met the performance criteria. For several of the syndromes, this process required injection of more than 20 tests orders, especially for detection of the gradual signals.

The Detection Performance columns of Table 2 provide sensitivity and recurrence metrics for each selected algorithm, with details for each syndrome and signal type. For each syndrome/signal type combination, we conducted sets of 1000 simulation trials using injected signals calculated for a fixed number of added cases in each trial. From the tabulated algorithm outputs, we calculated sensitivity for each candidate alerting threshold as the number of detected events (algorithm value > threshold) divided by the number of trials (= 1000). We calculated recurrence as the mean number of weeks between alerts. Beginning with total injection counts of only 5 cases, we repeated these sets of trials with higher case counts (i.e. stronger injected signals) until at least one candidate algorithm achieved the required 90% sensitivity and minimum 8-week recurrence. Table 2 shows the minimal injection case counts and the algorithm thresholds needed to meet these requirements for the selected algorithms.

The reader should not infer from Table 2 that the selected algorithms would give 95% sensitivity to any outbreak affecting the chosen syndrome groups, even when restricted to horses in Colorado. Outbreak sizes and durations cannot be predicted, and few are documented with population-level data in the literature. Our injected signals were based on the advice of APHIS veterinary epidemiologists and those they consulted, and we compared algorithms by increasing the injected signal strength until the desired sensitivity and alert burden were met. Separate studies are required to determine the expected sensitivity and alert rates for outbreaks of specified size and temporal progression.

**Fig 3:**
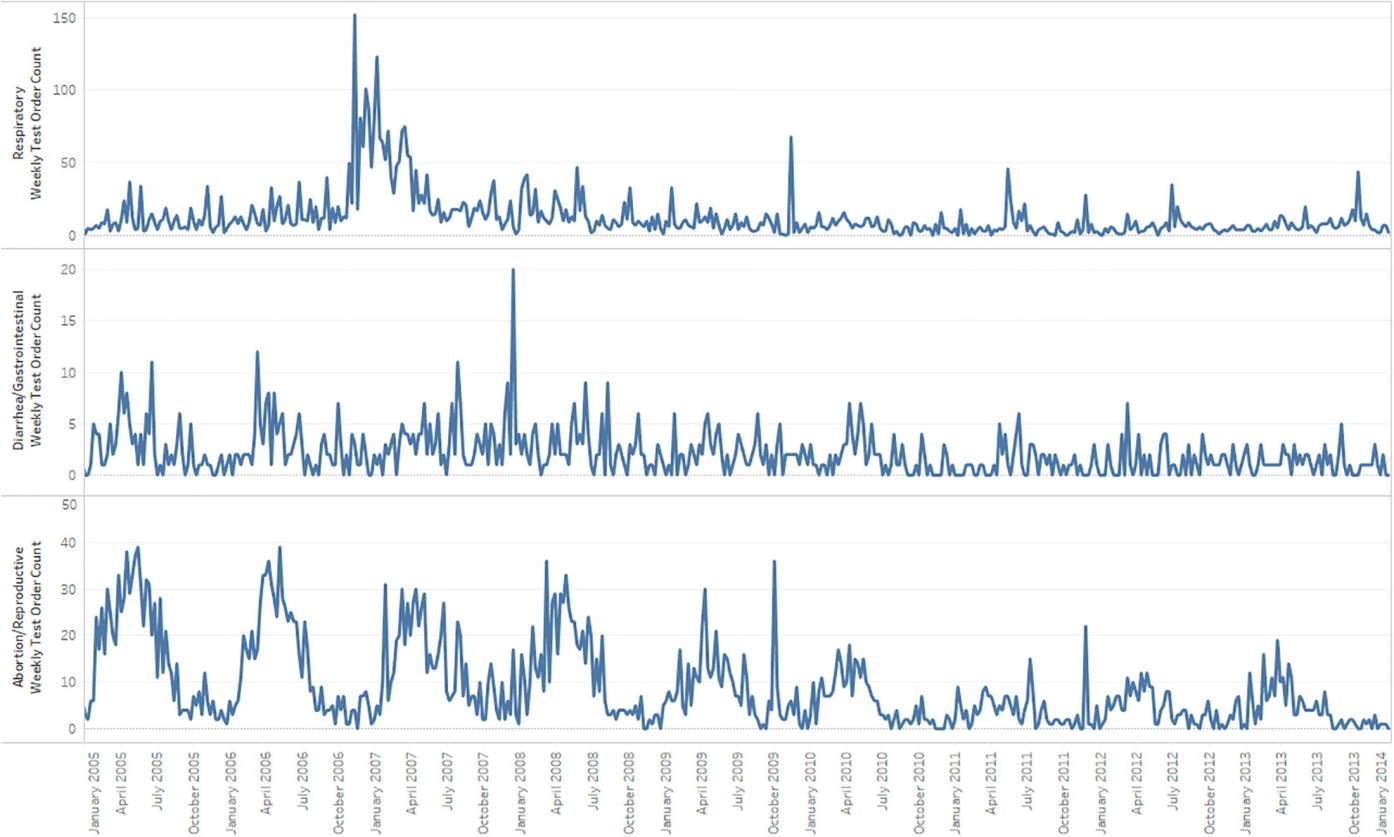
Time series plots of weekly counts of Abortion/Reproductive, Diarrhea/GI, and respiratory syndromes.

**Fig 4:**
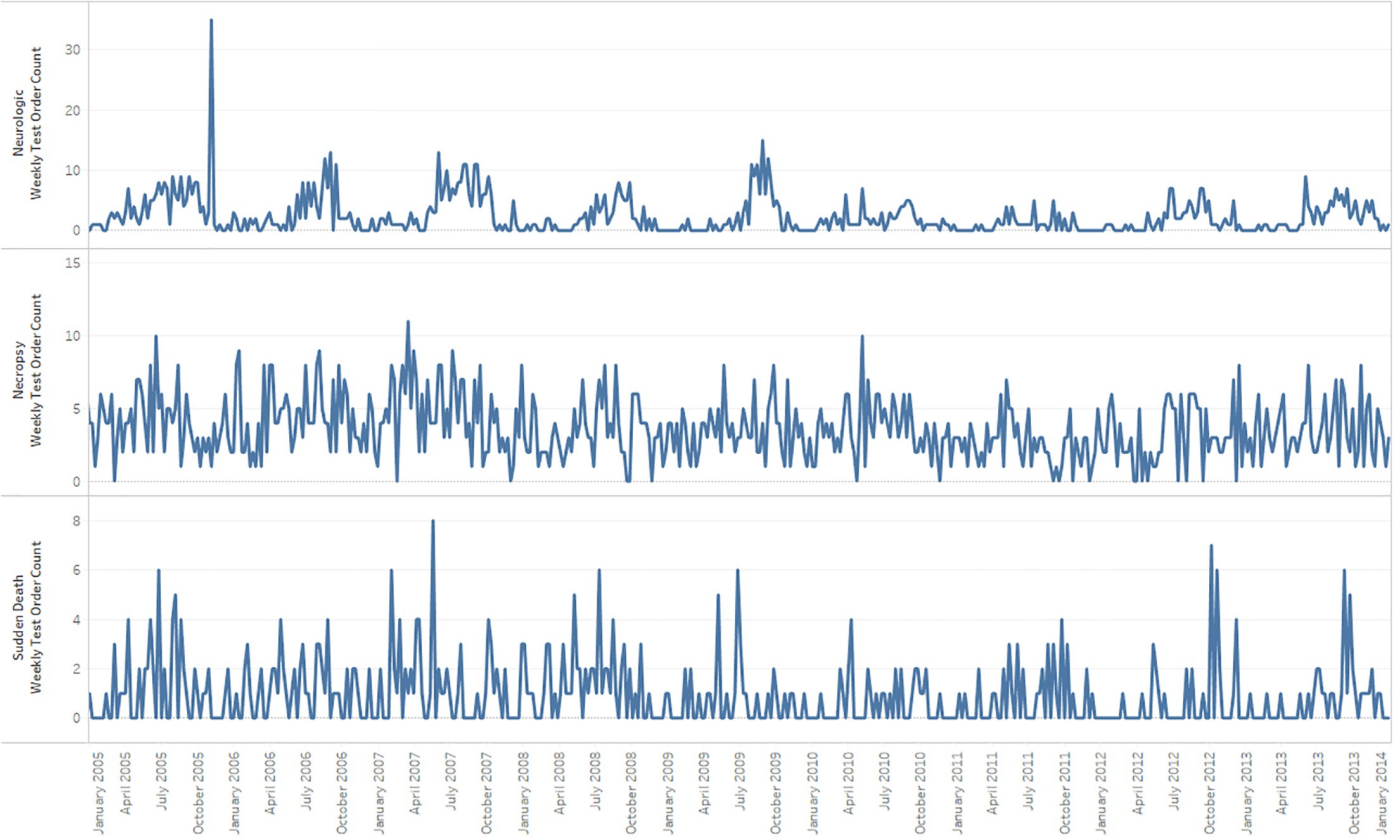
Time series plots of weekly counts of Neurologic, necropsy, and sudden death syndromes.

For visual context, Figs 3 and 4 present weekly time series covering nearly 11 years for six syndromes. Seasonal behavior is evident in the Abortion/Repro syndrome and less clearly in the Diarrhea/GI and Neurological syndromes. However, the seasonal behavior lacks the consistency that might be evident for other species and for larger animal populations, and this inconsistency likely explains why the historical limits method did not give superior performance when 1000 outbreak signals were injected randomly over the data interval in repeated trials. For nation-level datasets or for bovine or other species, the relative performance of seasonally adjusted methods may improve on richer, more structured time series, and regression methods may be applicable and provide more improvement. For the syndromes such as the neurological and sudden death, with median counts below two orders per week, performance improvements beyond the adaptive control chart-type methods used here will be difficult to achieve with sophisticated models.

For four of the syndromes, note that only ten injected cases were required to achieve this detection capability for the most efficient algorithms. However, for the respiratory syndrome with a standard deviation of over 14 test orders per week and target signals spread over six weeks, a total of 100 injected cases were required to attain 90% sensitivity with a manageable alert burden. In summary, the table recommendations are determined by large sets of simulated runs constrained by the historical syndrome incidence patterns, by the signal types of interest, and by the sensitivity and alert rate requirements.

## Discussion

This manuscript provides a detailed example of the process of understanding and transforming complex laboratory data into quantified relevant animal health indicators that can be routinely monitored for situational awareness and for early notification of potential health threats. Central questions are:

1. What is the case unit of interest?
  Deduplication procedures may be nontrivial, such as determining whether it is possible to consider each animal as a separate case. Counting each horse as a case depends on the ability to uniquely identify each horse with reasonable confidence. The horse’s name specified within each submission was used to detect each unique case. The breed, age, sex, and other individual attributes were not used. The alternative option is to consider all animals included in the entire submission as an animal health event, or case, representing one or more horses. This decision is essential to determining surveillance time series and baseline values that yield useful, unbiased trends reflecting current infectious disease processes.
2. Which of the tests are relevant to case identification, and which represent mandatory testing programs or other types of testing not reflective of animal disease syndromes of concern?
  Generally, information systems used for passive surveillance were not designed for that purpose. The system that is the source of CSU VDL test orders includes orders not only for testing of currently sick animals, but also for national, state, or local surveillance programs or other types of testing not reflective of transmissible animal disease syndromes of concern. Some examples include Coggins testing in horses or weekly fecal testing in all hospitalized equine patients. Including this testing may result in alerts reflecting batches of tests that are regulatory, seasonal, routine, or otherwise not indicative of a veterinarian’s concern for sick horses. Such alerts are of no epidemiological interest and may mask trends that do indicate such concerns. If working with data collected in a LIMS that does not explicitly represent testing performed for mandatory reasons or for routine animal health management, it may be necessary to explore unexpected testing frequencies and work with subject matter experts in order to identify testing not relevant to case identification. Identifying mandatory or routine tests was not trivial in the current project and required multiple discussions with laboratorians.
3. Given the concerns and resources of the monitoring agency, which data fields are useful for case definition rules?
  For practical classification of complex lab data, we sought the simplest possible criteria sufficient to distinguish disease threats of interest. The test battery name alone was sufficient when available, though the battery field was completed in only 4% of the dataset accessions. As explained above, the test order name was sufficient for most orders without a battery name, and for the remaining 25% of orders, we considered the combination of test order name and specimen type sufficient for classification. As discussed, these rules were not perfect, and with no efficient way to validate precisely, final decisions were based on veterinary and laboratory experience.
  The fields chosen should be checked for completeness and uniform usage. In our application, multiple representations of the same specimen type and misspellings required pre-processing steps and repeated inspection. The use of free-text and pick lists may not be uniform among all data providers or over time, and such issues must be taken into account.
  Based upon further experience with laboratory findings for additional animal taxonomy groups (e.g. cattle, swine, poultry), along with testing data provided across multiple laboratories, it has become apparent that the use of terminology standards would improve the efficiency of the design of new lab-based syndromic surveillance systems (both for animal and human health monitoring). The formation and refinement of case definition rules based on testing orders and specimen types can be supported by following suggested guidance on the usage of terminology standards [15] (especially with syndrome mapping efforts across laboratories) along with sharing standardized case definition rules across animal health surveillance agencies.
4. What syndrome categories are most useful for routine monitoring?
  The veterinary epidemiologists that defined the syndromes in this project mainly sought logical categories based on body systems (abortion/reproductive, respiratory, gastrointestinal, neurological). Separate syndromes were also defined for diseases not exclusively associated with distinct body systems (systemic fungal, tickborne) and for unanticipated threat types that could be missed because of unknown causes and multiple clinical signs (sudden death, necropsy).
  The number of syndromes to monitor is a practical decision depending on the specificity of the data, disease threats of interest, and monitoring resources of the users. In the current application, additional data fields and more investigation capability may have made more and sharper syndrome categories practical.
5. How should diagnostic laboratory tests be applied in case definitions for operational surveillance?
  The rules used to identify syndrome cases directly affect the sensitivity and specificity of threat detection. In the equine surveillance application, test orders mapped to a syndrome were those likely performed for a horse with a current illness affecting the corresponding body system. The intent was to maximize the “signal” covered by included tests without losing it in the “noise” of regulatory and routine testing. The hierarchical rules were intended to capture these orders as efficiently as possible given the information in each order, and plausible causes of misclassification were discussed along with the rules.
  Formation of these case definition rules was mainly driven by veterinary epidemiologist knowledge in consultation with the CSU laboratorians. The data analysis was employed not to derive the classifications, but for testing and confirmation using the few known outbreak signals in the data.
  The syndromic surveillance system placed in operation over a period of 3 years (2016–2018) provided experience and further insight into the usefulness of the lab tests selected for detecting syndromes of interest. The tests identified as the top two most commonly performed for each syndrome (Table 1) accounted for the detection of cases that contributed to 66% of the alerts reported (29/44) over this 3 year period. Knowledge about the tests most frequently performed along with specialized insight into the frequency of endemic disease agents contributed to the level of effort dedicated to alert follow-up. Additionally, following two years of operation, it was determined that the occurrence of testing for selenium did not serve as a useful indicator prompting investigation into the frequency of sudden death in horses. Therefore, this test was removed from the detection of sudden death syndromic cases.
  Other researchers have applied machine learning techniques to develop lab testing [2] and necropsy findings case definitions [4] for surveillance in the animal health monitoring context. Some researchers have applied similar techniques more generally in monitoring human care-seeking behavior [16–18]. Developers of future systems, including for broader applications envisioned within USDA CEAH, may consider these techniques. In a published comparison testing several machine learning methods, Dorea et al. achieved best performance with a rule-based approach [2].
6. Once the syndromes are chosen, what alerting method(s) should be used to automate the recognition of potential problems for investigation or tracking?
  The importance of automated alerting algorithms is that data inspection is tedious and time-consuming, especially in view of the rapidly increasing set of data sources, and algorithms can filter out systematic data behavior such as annual or weekly cyclic patterns and issue alerts to draw attention to potential outbreak signals. The candidate algorithms, influenced by the data history and varied behaviors of the weekly syndromic time series, were chosen to provide high sensitivity and manageable alert burden for the RI group in extending their weekly monitoring to equine laboratory test orders. Discussions of target diseases and the specification of target signal types by the epidemiologists for the separate syndromes enabled the sets of Monte Carlo trials used to derive the preferred methods and settings. The approach for determining these preferences could be useful for developing similar systems, but resulting preferences will depend on monitoring needs and available data.

## Conclusions

The development of robust and relevant automated systems using veterinary laboratory data for monitoring animal health requires multiple tasks involving surveillance requirements assessment, operations and data analysis, adaptation of statistical alerting algorithms, and sustainable and reliable technology for acquiring and processing data and displaying the results.

The information available electronically supports tracking of a limited number of health threat types. Understanding of laboratory submission sources, laboratory workflow, data management and of syndrome-related outcomes are crucial to form syndrome groups for routine monitoring without artifactual alerting. This combined insight requires consultation among epidemiologists, laboratorians, veterinarians, and statistical analysts. For example, discovery of the set of testing batteries available for ordering would need to be performed separately for each laboratory. This would include learning the names used, naming standards, along with the set of tests triggered for each battery. It is hoped that the principal components and tasks used for building the lab order syndromic surveillance system described in this document provide a general framework for developing other similar systems.

In the current application involving equine health surveillance, effective and efficient choices for alerting methods and settings varied by syndrome and depended strongly on epidemiologist-specified performance requirements. Validation of the syndromes and method decisions was based on analysis of historical data and requires prospective experience for evaluation and adjustment.

## Supporting information

S1 Table Test order syndrome mapping table. (DOCX)

S2 Table Specimen type categories. (DOCX)

S3 Table Time series of weekly counts of equine syndrome groups used for algorithm evaluation. (XLSX)
