# Supplementary material for: Equine syndromic surveillance in Colorado using veterinary laboratory testing order data
Source: PLoS One. 2019 Mar 1;14(3):e0211335. doi: 10.1371/journal.pone.0211335 (PMC6396905; doi:10.1371/journal.pone.0211335)
Supplement: S1 Table — (DOCX) [file pone.0211335.s001.docx]

# S1 Table: Test Order Syndrome Mapping Table

| Syndrome | Test Name | Specimen Body System | Specimen Category |
| --- | --- | --- | --- |
| Abortion/Repro | Leptospirosis Canicola MAT |  |  |
|  | Lepto 5 MAT panel |  |  |
|  | Lepto Grippotyphosa MAT Serology |  |  |
|  | Aerobic & Anaerobic Culture - Food Animal | Reproductive |  |
|  | Lepto Hardjo MAT Serology |  |  |
|  | Lepto Icterohaemorrhagiae MAT Serology |  |  |
|  | Equine Herpesvirus-3 (EHV-3) - PCR |  |  |
|  | Equine Herpes Virus-1 (EHV-1) FA |  | fetal tissue |
|  | Equine Herpes Virus-3 (EHV-3) SN |  |  |
|  | Lepto Pomona MAT Serology |  |  |
|  | Aerobic & Anaerobic Culture | Reproductive |  |
|  | Equine Herpesvirus-1/4 (EHV-1/4) PCR |  | fetal tissue |
|  | Aerobic Culture | Reproductive |  |
|  | Equine Herpesvirus-1/4 (EHV-1/4)conv/real-time PCR |  | fetal tissue |
|  | Endometrial Biopsy |  |  |
|  | Equine Viral Arteritis (EVA) - PCR |  | Not(serum) AND Not(blood) AND Not(nasal swab) |
|  | Fungal Culture | Reproductive |  |
|  | Stain - Warthin Starry |  |  |
|  | Stain - IHC Leptospirosis |  |  |
|  | VTH - Aerobic Culture & Sensitivity | Reproductive |  |
|  | Abortion Screen |  |  |
|  | VTH - Anaerobic & aerobic Culture and Sensitivity | Reproductive |  |
|  | Leptospirosis - PCR |  |  |
| Diarrhea/GI | Rotavirus ELISA |  |  |
|  | Cryptosporidium Small Animal Acid Fast |  |  |
|  | Clostridial difficile A/B Toxin |  |  |
|  | Clostridium Fecal Culture |  |  |
|  | Internal Parasite | Digestive |  |
|  | Clostridium Perfringens Toxin ELISA |  |  |
|  | Cryptosporidium/Giardia IFA |  |  |
|  | Diarrhea Screen |  |  |
|  | Fecal Screen - PSPanel |  |  |
|  | Occult Blood |  |  |
| Necropsies | Necropsy VTH(Equine) |  |  |
|  | Necropsy Histopathology |  |  |
|  | Necropsy & Histo VTH (Equine) |  |  |
|  | Necropsy Equine Gross Examination Only |  |  |
|  | Necropsy (INSURANCE/LEGAL) |  |  |
| Neurologic | Aerobic & Anaerobic Culture - Food Animal | Nervous |  |
|  | Equine Herpes Virus-1 (EHV-1) SN |  | csf |
|  | Rabies FA |  |  |
|  | CSF Analysis w/ Cytology |  |  |
|  | Equine Herpes Virus-4 (EHV-4) SN |  | csf |
|  | Equine Herpesvirus-1/4 (EHV-1/4) PCR | Nervous |  |
|  | Equine Herpesvirus-1/4 (EHV-1/4)conv/real-time PCR | Nervous |  |
|  | Equine West Nile Virus (WNV) IgM |  |  |
|  | Aerobic Culture | Nervous |  |
|  | Equine Encephalitis - NVSL |  |  |
|  | Fungal Culture | Nervous |  |
|  | Equine Herpes Virus-1 (EHV-1) FA | Nervous |  |
|  | Aerobic & Anaerobic Culture | Nervous |  |
|  | Western Equine Encephalitis Virus (WEEV) - PCR |  |  |
|  | West Nile Virus (WNV) - PCR |  |  |
|  | VTH - Aerobic Culture & Sensitivity | Nervous |  |
|  | VTH - Anaerobic & aerobic Culture and Sensitivity | Nervous |  |
| Respiratory | Baermann |  |  |
|  | Aspergillis AGID |  |  |
|  | Aerobic Culture | Respiratory |  |
|  | Cytology -Additional site | Respiratory |  |
|  | Cytology -Aspirate/Impression Smear | Respiratory |  |
|  | Aerobic & Anaerobic Culture - Food Animal | Respiratory |  |
|  | Aerobic & Anaerobic Culture | Respiratory |  |
| Respiratory | Chlamydophila (Companion Animal) - PCR |  |  |
|  | Fungal Culture | Respiratory |  |
|  | Streptococcus equi - PCR |  |  |
|  | VTH - Aerobic Culture & Sensitivity | Respiratory |  |
|  | VTH - Anaerobic & aerobic Culture and Sensitivity | Respiratory |  |
|  | Equine Herpes Virus-4 (EHV-4) SN |  | serum |
|  | Influenza A Virus - PCR |  |  |
|  | Equine Herpes Virus-1 (EHV-1) SN |  | serum |
|  | Equine Viral Arteritis (EVA) SN |  |  |
|  | Equine Viral Arteritis (EVA) Screen (SN) |  |  |
|  | Equine Viral Arteritis (EVA) - PCR |  | serum OR blood OR nasal swab |
|  | Equine Influenza - HI |  |  |
|  | Equine Herpesvirus-1/4 (EHV-1/4)conv/real-time PCR | Not(Nervous) | Not(fetal tissue) |
|  | Equine Herpesvirus-1/4 (EHV-1/4) PCR | Not(Nervous) | Not(fetal tissue) |
| Sudden Death | Bacillus anthracis (Anthrax) real-time PCR |  |  |
|  | Selenium (Hydride FAAS) |  |  |
| Systemic Fungal | Aspergillis AGID |  |  |
|  | VTH - Stain - GMS |  |  |
|  | Stain - GMS |  |  |
|  | Fungal Culture |  | Not(fungal skin) |
| Tickborne | Tick Panel-Ehrlichia Anaplasma Lyme ELISA |  |  |
|  | Ehrlichia spp/Anaplasma/Neorickettsia/Wolbachia - PCR |  |  |
