# Supplementary material for: Equine syndromic surveillance in Colorado using veterinary laboratory testing order data
Source: PLoS One. 2019 Mar 1;14(3):e0211335. doi: 10.1371/journal.pone.0211335 (PMC6396905; doi:10.1371/journal.pone.0211335)
Supplement: S2 Table — (DOCX) [file pone.0211335.s002.docx]

## Specimen Type Categories

| **serum category** | |
| --- | --- |
| **Specimen Types** | |
| Blood & Serum | Acute Serum |
| Blood & Serum & Urine | Serum |
| Blood and Serum | Serum - Acute |
| Blood, Serum | Serum - Convalescent |
| Blood, Serum, Urine | Serum - RBTT |
| Blood/Serum | Serum - SST |
| Blood/Serum/Urine | serum & blood |
|  | Serum/Blood |

| **blood category** | |
| --- | --- |
| **Specimen Types** | |
| Blood | Nasal Swab & EDTA Whole Blood |
| Blood & Serum | serum & blood |
| Blood & Serum & Urine | Serum/Blood |
| Blood and Serum | Whole Blood |
| Blood, EDTA, LTT | Whole Blood , EDTA |
| Blood, Heparinized. GTT | Whole Blood , EDTA, PTT |
| Blood, Serum |  |
| Blood, Serum, Urine |  |
| Blood/Serum |  |
| Blood/Serum/Urine |  |

| **nasal swab category** | |
| --- | --- |
| **Specimen Types** | |
| nasal swab | L&R Nasal swab |
| Nasal & Abscess swabs per Vet | pus/nasal discharge |
| Nasal Swab | Swab - Nasal |
| Nasal Swab & EDTA Whole Blood | Swab - Nasal Wash |
| Nasal swabs pooled | Swab - Nasopharyngeal |

| **csf category** | | |
| --- | --- | --- |
| **Specimen Types** | | |
| CSF | Cerebral Spinal Fluid | Spinal fluid |

| **fetal tissue category** | |
| --- | --- |
| **Specimen Types** | |
| Liver | Placenta |
| Liver Tissue | pool |
| Liver, Lung, Kidney, Placenta | Pool Lung/Liver/Plac./Kidney |
| liver, lung, placenta, kidney | POOL= Lung, Kidney, Placenta |
| Liver, lung, placenta, kidney, spleen tissue | Pool=Kidney,Liver |
| Liver/Kidney/Lung/placenta Pool | Pooled lung, liver & kidney |
| Lung | Pooled spleen/liver/kidney |
| lung liver | Pooled tissues |
| Lung Tissue | Pooled=Liver, Kidney |
| Lung, kidney, liver | Pooled=Lung, Liver, Spleen |
| Lung, Kidney, Placenta pool | Spleen Tissue |
| Lung, Liver, Placenta pool | Tissue |
| Lung, Placenta, Kidney | Tissue pool |
| Lung, placenta, kidney tissue | Tissue Collection |
| Lung/ kidney/ placenta pool | tissue pool |
| Lung/Kidney/Placenta | FFPE Tissue |
| Lung/kidney/placenta pool | Kidney Tissue |
| Lung/Liver/Kidney/Placenta |  |
| Lung/liver/kidney/placenta pool |  |
| Lung/liver/placenta |  |
| Lung/liver/placenta pool |  |

| **fungal skin category** | |
| --- | --- |
| **Specimen Types** | |
| scabs hair | Skin scraping |
| Chest wound swab | Skin Scraping / Hair |
| Hair | Skin Swab |
| Scabs and Hair | Skin swab from muzzle |
| scabs hair | Skin Tissue |
| Skin | Swab |
| Skin Biopsy (Neck) | Swab - Skin Wound |
| skin crust right stifle and inguinal area/swab | Swab - Wound |
| Skin dist limb | Wound Swab |
| Skin lesion | WOUND-SWAB |
| Skin lesions |  |
| Skin Punch |  |

## Specimen Type Body Systems

| **Digestive body system** | |
| --- | --- |
| **Specimen Types** | |
| Cecal Contents | Intestinal Tissue |
| Cecum | Intestine |
| Colon | Jejunum |
| Colon Contents | Large colon |
| Colon Tissue | Large Intestinal Contents |
| Diarrhea Swab | Pool feces |
| Duodenal Contents | Rectal swab |
| Duodenum | Reflux |
| Duodenum Tissue | Small Intestinal Tissue |
| Fecal Smear | small intestine |
| Fecal Swab | St Ct |
| Feces | Stcont |
| Gastric Contents | Stct |
| Gastric Fluid | Stom cnt |
| Gastric Reflux | stomach cont |
| Gastrointestinal Contents | Stomach Content |
| GI content | Stomach contents |
| GI/Stct | Stomach Swab |
| Intestinal Contents |  |

| **Nervous body system** |
| --- |
| **Specimen Types** |
| brain |
| Brain Swab |
| Brain Tissue |
| Brainstem Tissue |
| Cerebellum and Brainstem |
| Cerebral Spinal Fluid |
| CSF |
| Meningeal Swab |
| Spinal Cord Tissue |
| Spinal fluid |

| **Reproductive body system** | |
| --- | --- |
| **Specimen Types** | |
| Cervical mass | Placental tissue swab |
| Cervical Swab | R Clitoral Sinus |
| cervix | Slide, Uterine Cytology |
| Cervix Swab | uterine |
| Cervix/Endometrium | Uterine Biopsy |
| Clit fossa | Uterine culture |
| Clit sinus | Uterine Culture Swab |
| Clit Sinuses | Uterine Fluid |
| Clitoral central fossa | Uterine lavage |
| Clitoral sinuses | Uterine Lavage Fluid |
| Clittoral Fossa | Uterine Slide |
| Cytology smear, uterine | Uterine Slides |
| Ejaculate Swab | Uterine Swab |
| Endometrial Fld Swab | Uterine Swab Pool |
| Endometrial Swab | Uterine Tissue |
| Endometrium | Uterine/slide |
| Fetus | uterine/swab |
| FNA Penile | Uterus |
| Glans | Uterus Swab |
| L Clitoral Sinus | Uterus Tissue |
| Penile | uterus/swab |
| Penile Lesion Swab | vagina |
| Placenta | Vaginal swab |
| Placenta/Uterine fluid | Vulva Swab |
| Placental Swab | Endometrial Biopsy |

| **Respiratory body system** | |
| --- | --- |
| **Specimen Types** | |
| Fluid from TTW | Pharyngeal Swab |
| g-pouch | Pleural |
| Gutteral pouch | Pleural Fluid |
| Gutteral Pouch Aspirate | Pleural fluid - left |
| gutteral pouch lavage | Pleural fluid - right |
| Gutteral pouch wash L | Plural fluid |
| Gutteral pouch wash R | pooled sinus discharge |
| GUTTORAL POUCH | pus/nasal discharge |
| Guttural Pouch | R nasal |
| Guttural pouch fluid | R Thoracic fluid |
| Guttural pouch wash | Rostral maxillary sinus |
| L Thoracic fluid | Swab - Nasal |
| L&R Nasal swab | Swab - Nasal Wash |
| lft side gutteral pouch fluid/2 samples pooled | Swab - Nasopharyngeal |
| Lung | Thoracic fluid |
| Lung Abscess | Thoracic L |
| Lung Fluid | Thoracic R |
| Lung swab | Throat Swab |
| Lung Tissue | Trach |
| Maxillary nasal sinus | Trach fluid |
| Maxillary sinus | Trach fluid swab |
| maxillary sinus swab | Trach wash |
| Nasal | Trach wash/guttural pouch wash pool |
| nasal swab | Tracheal |
| Nasal & Abscess swabs per Vet | Tracheal Fluid |
| Nasal Cyst Fluid | Tracheal Swab |
| Nasal Discharge | Tracheal wash |
| Nasal Mass | trans trach aspir |
| Nasal passage | TRANS TRACHEAL WASH |
| Nasal sinus pus | transtracheal wash |
| Nasal Swab | Transtracheal Wash Fluid |
| Nasal Swab & EDTA Whole Blood | TTA |
| Nasal swabs pooled | TTF |
| Nasal wash | TTW |
| Nasal/gut pouch | ttw fluid |
| Nasopharyngeal | TTW swab |
| Nose | TTW/fluid |
| nostril | TTW/swab |
